# *Safety I* to *Safety II*: A Paradigm Shift or More Work as Imagined? Comment on "False Dawns and New Horizons in Patient Safety Research and Practice"

**DOI:** 10.15171/ijhpm.2018.24

**Published:** 2018-03-07

**Authors:** Kelly M. Smith, Annette L. Valenta

**Affiliations:** ^1^MedStar Institute for Quality and Safety, MedStar Health, Columbia, MD, USA.; ^2^Department of Medical Education, College of Medicine, University of Illinois at Chicago, Chicago, IL, USA.

**Keywords:** Patient Safety, Quality Improvement, Professionalism, Education

## Abstract

In their editorial, Mannion and Braithwaite contend that the approach to solving the problem of unsafe care, *Safety I*, is flawed and requires a shift in thinking to what they are calling *Safety II*. We have reservations as to whether by itself the shift from *Safety I* to *Safety II* is sufficient. Perhaps our failure to improve outcomes in the field of patient safety and quality lies less in our approach – *Safety I* vs. *Safety II* – and more in the lack of an agreed upon, commonly understood set of core competencies (knowledge, skills, and attitudes) needed in its workforce. The authors explore in this commentary the need to establish core competencies as part of the pathway to professionalism for the discipline of patient safety and quality.

We read with great interest the provocative editorial by Mannion and Braithwaite, *False Dawns and New Horizons in Patient Safety Research and Practice*.^1^ In their article, the authors developed a sound argument underlying the intractability of improvements in patient safety. As many as one in ten patients is harmed by health-related care, indicating a principal problem within health systems around the globe.^1,2^ Mannion and Braithwaite contend that our past approach to solving the problem of unsafe care, *Safety I*, is fundamentally flawed, requiring a paradigm shift of thinking towards what they are calling *Safety II*. *Safety I*, the approach adopted by most healthcare systems worldwide, focuses on understanding why a patient safety event has occurred using standard approaches (eg, root cause analysis, incident reporting, failure modes and effects analysis) adapted for use in healthcare from other high reliability industries.^1,3-5^ Our concentration of resources on rare events, linear causality, and individual culpability has resulted in minimal gains towards building safer systems since the publication of *To Err is Human* launched the modern-day patient safety movement.^6^ The driving question is whether the *Safety II* approach will produce different results by combining *Safety I* thinking with examination of the how and why of safely delivered care? Or, alternatively, is *Safety II* yet another case of “*work as imagined*”?^7^

We have reservations as to whether by itself the shift from *Safety I* to *Safety II* is sufficient to disrupt the intractability in the fields of patient safety practice and research. The underlying hypothesis of Mannion and Braithwaite is predicated on the assumption that systems thinking is the pervasive approach to examining patient safety incidents and that *Safety II* builds upon the foundational competencies (knowledge, skills, and attitudes) developed through the systematic and routine application of the principles of *Safety I*. It is here that we pause and suggest that their hypothesis may not accurately reflect patient safety as practiced in most healthcare settings, or better said the “*work as done.*”^7^ We offer an alternative theory for consideration.

## Patient Safety in Practice: A Well-Intentioned Workforce

Perhaps our failure to improve outcomes in the field of patient safety and quality lies less in our approach – *Safety I*vs. *Safety II* – and more in the lack of an agreed upon, commonly understood set of core competencies (knowledge, skills, and attitudes) needed in its workforce. Those currently doing the work of patient safety and quality are formally trained in medicine, nursing, law, pharmacy, and healthcare administration, and not formally trained in the emerging profession of patient safety and quality itself. Before we decide “*to throw the baby out with the bathwater,*”^8^ perhaps we need to broaden our perspective on the problem.

Our experience as patient safety scientists, educators, and practitioners has fostered an appreciation for the challenges faced by other patient safety practitioners at the sharp end of their work. What we have found is an incredibly dedicated and passionate group of individuals, drawn to solve the problems in patient safety and quality, who come to the field from disparate professions, education, and training. Many patient safety practitioners lack the essential competencies (knowledge, skills, and attitudes) needed to further the modern-day patient safety agenda.^9-12^ As an example, one core competency to foster successful application of *Safety I* principles is the ability to conduct comprehensive, systems-based, root cause analyses (RCA) and to develop and implement sustainable plans of action.^9,12^ A recent study examining 302 RCAs concluded that the proposed solutions resulting from the RCA were less likely to decrease event recurrence and less likely to recommend actions to improve the RCA processes and local implementation.^13,14^ Few leaders in quality and patient safety receive formal training to perform the technical work within the profession. We contend that these patient safety leaders, those responsible for conducting the technical work of patient safety, require formal education in the core competencies^9,12^ to perform the *imagined work* as designed to achieve the aims of *Safety I*. Until the field of patient safety unites around these competencies and requires certification as a profession, neither *Safety I* nor *Safety II* will reach its full potential to eliminate preventable harm due to health-related care.

## Scholarship as a Competence in Patient Safety

It was only five years ago that the term “*Patient Safety*” was added to the medical subject headings (MeSH) vocabulary thesaurus, allowing for increased specificity in searching published literature through the U.S. National Library of Medicine.^15^ Safety science is still relatively new, building on the longstanding fields of ergonomics, human factors engineering, sociology, and anthropology to name a few. Applying the science of these fields to the complex and adaptive systems that comprise healthcare is neither intuitive nor direct. It requires not only a specialist knowledge of an individual field, but a generalist knowledge of complementary fields of study. Thus, as part of formal training and certification as professionals in the field, future practitioners in patient safety and quality must demonstrate the ability to work comfortably in a world of applied scholarship. By necessity, safety science often diverges from traditional science because frequently the problem at hand cannot be tested using hypothesis-driven, randomized controlled trials; however, since we are talking about human behavior within complex systems, qualitative and quasi-experimental research designs are of great importance within improvement efforts. As suggested by Mannion and Braithwaite:

*“If we want to achieve different results then we need to be less reverential towards the orthodox paradigm, get beyond simplistic system thinking, expand our research horizons, and advance new and better ways for understanding and intervening in patient safety.”* ^1^

Transformation of the profession of patient safety as an independent research path will require more than just the change in our approach to study proposed by Mannion and Braithwaite (shifting focus from what goes wrong to also studying what is going right), but will also require a change in how we train future scholars.

## What is Next? Patient Safety as a Profession

Today, the role of a patient safety leader is often garnered through promotion, not through evidence of advanced training and application of knowledge to practice. Every health profession today has built aspects of patient safety education, from its uniprofessional perspective, within the discipline’s curriculum. What is missing, however, is a recognized professional trained in the competencies of patient safety and quality to *lead* the patient safety agenda within organizations and systems. Ensuring that patient safety leaders become recognized, visible, and have the authority to lead the technical work of the profession of patient safety, requires that we come to consensus on that set of competencies to establish the profession of patient safety.

In order for us to reverse the apparently intractable state of patient safety and quality improvement, those disciplines drawn to the work, and the healthcare institutions employing them, must recognize patient safety and quality as a profession, “a calling requiring specialized knowledge and often long and intensive academic preparation.”^16^ In their 1996 Technical Report, Ford and Gibbs describe “A Model of a Profession” and its accompanying infrastructure. The components of a profession include initial professional education, accreditation, skills development, certification or licensure, professional development, a code of ethics, and professional societies.^17^ Toward that end, nine graduate programs from leading universities in the United States and Canada have begun the process with the Commission on Accreditation of Healthcare Management Education (CAHME) to establish accreditation standards for graduate programs in patient safety and quality. One component of the standards is establishing the core competencies required for students graduating with a degree in patient safety and quality at the Master’s level.

Until the global community calls for and drives the adoption of fundamental principles and core competencies for the profession of patient safety and quality, our performance of *Safety I* and *Safety II* will fall short, perpetuating existing patient safety practices*.*

## Ethical issues

Not applicable.

## Competing interests

Authors declare that they have no competing interests.

## Authors’ contributions

Both authors contributed equally to the writing of this article.

## Authors’ affiliations

^1^MedStar Institute for Quality and Safety, MedStar Health, Columbia, MD, USA. ^2^Department of Medical Education, College of Medicine, University of Illinois at Chicago, Chicago, IL, USA.
